# Dynamic changes in fecal bacterial microbiota of dairy cattle across the production line

**DOI:** 10.1186/s12866-022-02549-3

**Published:** 2022-05-14

**Authors:** Lei Zhao, Xunde Li, Edward R. Atwill, Sharif S. Aly, Deniece R. Williams, Zhengchang Su

**Affiliations:** 1 Department of Bioinformatics and Genomics, the University of North Carolina at Charlotte, Charlotte, North Carolina USA; 2 Western Institute for Food Safety and Security, University of California, Davis, California USA; 3 Department of Population Health and Reproduction, School of Veterinary Medicine, University of California, Davis, CA USA; 4 Veterinary Medicine Teaching and Research Center, School of Veterinary Medicine, University of California, Davis, CA USA

**Keywords:** Dairy cattle, Production line, Microbiome, Gastrointestinal, 16S rRNA, Bioinformatics

## Abstract

**Background:**

Microbiota play important roles in the gastrointestinal tract (GIT) of dairy cattle as the communities are responsible for host health, growth, and production performance. However, a systematic characterization and comparison of microbial communities in the GIT of cattle housed in different management units on a modern dairy farm are still lacking. We used 16S rRNA gene sequencing to evaluate the fecal bacterial communities of 90 dairy cattle housed in 12 distinctly defined management units on a modern dairy farm.

**Results:**

We found that cattle from management units 5, 6, 8, and 9 had similar bacterial communities while the other units showed varying levels of differences. Hutch calves had a dramatically different bacterial community than adult cattle, with at least 10 genera exclusively detected in their samples but not in non-neonatal cattle. Moreover, we compared fecal bacteria of cattle from every pair of the management units and detailed the number and relative abundance of the significantly differential genera. Lastly, we identified 181 pairs of strongly correlated taxa in the community, showing possible synergistic or antagonistic relationships.

**Conclusions:**

This study assesses the fecal microbiota of cattle from 12 distinctly defined management units along the production line on a California dairy farm. The results highlight the similarities and differences of fecal microbiota between cattle from each pair of the management units. Especially, the data indicate that the newborn calves host very different gut bacterial communities than non-neonatal cattle, while non-neonatal cattle adopt one of the two distinct types of gut bacterial communities with subtle differences among the management units. The gut microbial communities of dairy cattle change dramatically in bacterial abundances at different taxonomic levels along the production line. The findings provide a reference for research and practice in modern dairy farm management.

**Supplementary Information:**

The online version contains supplementary material available at 10.1186/s12866-022-02549-3.

## Background

It is now well-established that microbiota, especially bacterial communities, play crucial roles in the physiology and health of all mammals. While the number of bacterial cells in the human body has been estimated as 10 times more than the number of human cells [1] or at roughly the same order in a recent study [2], this ratio can rise to approximately 120 times in ruminants such as cattle [3]. Cattle depend on their gastrointestinal microbiota to digest and convert the plant mass that cannot be directly digested into absorbable nutrients necessary for host health and development. Thus, a better understanding of the structure of the gastrointestinal microbiota is instrumental for both production and scientific inquiry. Particularly, in the modern dairy system, calves, heifers, and cows at different ages and production stages are raised and managed in independent yet inter-connected management units with housing and dietary differences [4]. Specifically, male calves are commonly sold at birth while female calves are raised on the source dairy from birth to weaning (approximately 70 days of age) in group pens or more commonly in individual hutch units that may be wooden, metal, or plastic of combination of materials and where calves are fed milk two to three times a day and offered solid grain mix ad libitum with the goal of transitioning to a solid diet by the age of weaning. Once weaned, growing calves are moved to group pens where they are fed a roughage diet with concentrate formulated to their nutrient requirements until breeding age. Once bred, pregnant heifers are moved to maternity pens closer to their calving date. After calving, the newborn calf is fed colostrum and raised as described earlier. Post-partum cows are moved to the fresh cow milking pen before being moved to subsequent milking pens depending on the dairy’s management practices with respect to breeding (not pregnant and pregnant pens) and level of milk production (high and low milking pens). Late pregnant cows around 60 days prior to calving are dried off, an industry practice of cessation of milking to allow the dam to replenish her body resources and initiate colostrogenesis in preparation for giving birth to a calf. Adult cows at different stages are commonly fed a total mixed ration formulated to meet the specific stage of lactation nutrient requirements. Given such differences in management, housing, and diets, it is important to understand the dynamics of the gut microbiota changes between/among different management units over the entire production life cycle of dairy cattle. The knowledge gained can help design new strategies to improve production as well as the health of both the animals and humans who consume the produced meat and milk.

Since traditional culture-dependent techniques can only recover a small portion of the microbial population [5], several research groups have explored the gut microbiota of dairy cattle using the next-generation sequencing (NGS) technologies [3, 6–13]. For instance, Mao et al. analyzed the microbiota of ten gastrointestinal sites in Holstein cattle using 16S rRNA gene sequencing and found that these cattle hosted microbiota with significant spatial heterogeneity [7]. Dill-McFarland et al. analyzed the succession pattern of bacterial communities in dairy cattle from 2-week-old to first lactation [9]. Shanks et al. profiled the structure of fecal bacteria in cattle from various feeding operations [12]. However, a systematic contrast of the gut bacterial microbiota in dairy cattle along all the management units on a modern farm is still absent. To fill these gaps, we analyzed the gut bacterial communities of dairy cattle in management units of a dairy herd in California starting from the newborn to late lactation and dry cows using 16S rRNA gene sequencing data.

## Results

### Composition of the bacterial communities in the fecal samples

**Fig. 1:**
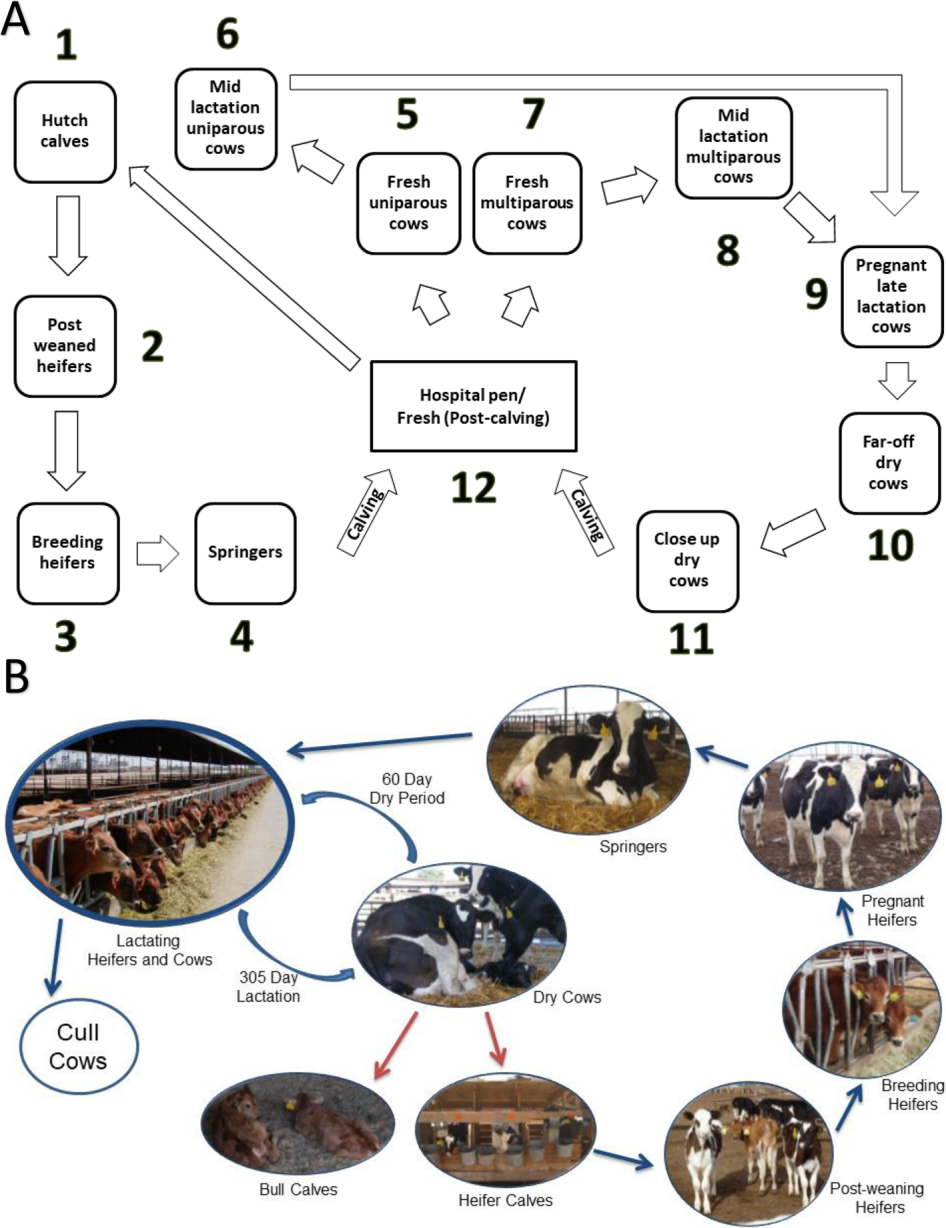
Management units and dairy cattle life cycle on a dairy farm. **A** Schematic diagram of the independent yet inter-connected management units of the dairy herd where the samples were collected. The number around each box represents the management unit ID used in this study. **B** Dairy cattle life cycle by age and production stage

We analyzed a total of 90 fecal samples collected on the same day from 90 different dairy cattle in 12 management units (Fig. 1, Table 1) in a dairy herd. We generated a total of 6,092,309 16S rRNA gene paired-end sequencing reads with an average of 67,692 ± 7963 reads per sample. Clustering sequence reads from V3/V4 regions of 16S rRNA genes into different Operational Taxonomy Units (OTUs) has been a widely adopted strategy. However, the traditional OTU methods obscure similar sequences by grouping them into a consensus sequence and fail to tell the technical errors from real biological variations [14]. Thus, we used a more recent method DADA2 that records how many times an exact sequence was read and infers sequence variants by a statistical model learning the sequencing error rate from the samples themselves [14]. Given the common usage of the term, we use OTU to represent the inferred sequence variant instead of Amplicon Sequence Variant (ASV) that the DADA2 authors proposed [15]. After quality filtering and chimera removal with DADA2 [14], a total of 5,080,044 sequence reads with an average of 56,444 ± 6633 reads per sample were preserved. OTUs that were taxonomically classified as “Archaea/Eukaryote” or appeared in less than 9 samples (10% of sample size) were excluded. Although the number of sequencing reads varied in our samples, rarefaction curves showed that our sampling was sufficient to represent the bacterial communities as the curve had almost plateaued or started to form a plateau (Additional file 1: Fig. S1). The OTU table was normalized by rarefying with a threshold of 12,637 sequences, which is the minimum number of reads of all the 90 samples after processing. All downstream analyses were based on the normalized OTU table (Additional file 2: Supplementary file 1).

**Table 1:** Classification of cattle based on the growth, production stages, and their residential management units of the dairy herd where samples were collected

Management Unit	No. of Samples	Unit ID	Description
Hutch calves	9	1	From birth to approximately 1–2 weeks after weaning, individually housed (1 to 70 days age approximately)
Post weaned heifers	6	2	Group-housed heifers and bull calves (if not sold) fed solid diet
Breeding heifers	8	3	Approximately 13 to 15 months old
Springer	8	4	Within 1 to 4 weeks from calving, pregnant nulliparous heifers
Fresh uniparous cows	8	5	1 to 2 months post-calving, first lactation cows
Mid-lactation uniparous cows	8	6	60 to 250 Days in Milk, first lactation
Fresh multiparous cows	8	7	1 to 2 months post-calving, second lactation or greater
Mid-lactation multiparous cows	8	8	60 to 250 Days in Milk, second lactation or greater
Pregnant late lactation cows	8	9	> 250 Days in Milk
Far-off dry cows	3	10	21 to 60 days prior to calving, multiparous dry cows
Close up dry cows	8	11	within 21 days prior to calving, multiparous dry cows
Hospital pen/ Fresh (post-calving)	8	12	Lactating cows treated with medication that requires milk withdrawal. May include fresh cows (post-calving) during their transition from producing colostrum to milk and/or pending milk withdrawal after treatment at drying (prior to entering Far-off dry pen).

**Fig. 2:**
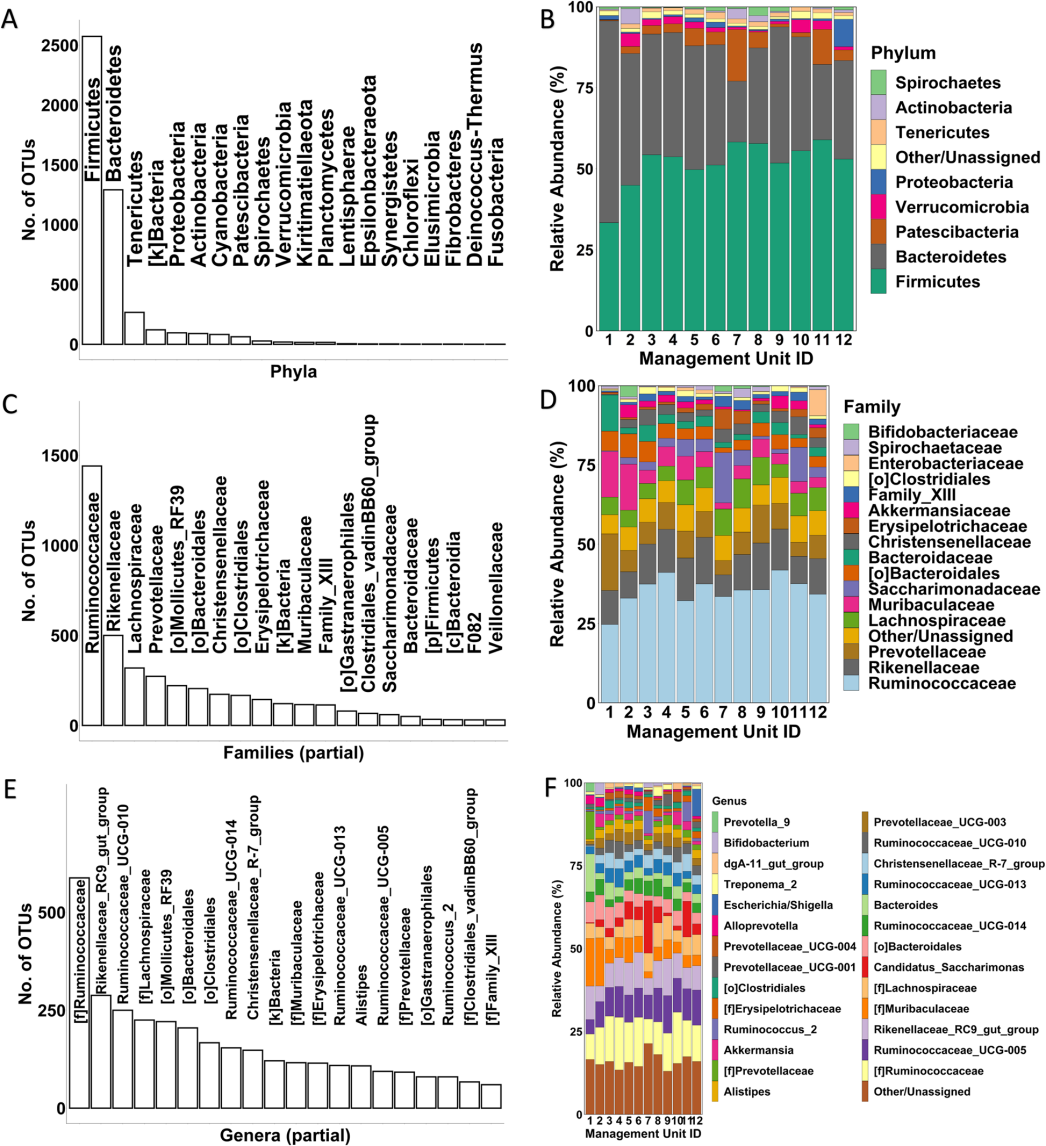
Assignment of the OTUs to different taxonomic levels. **A** The number of OTUs assigned to different phyla. **B** Average relative abundances of the phyla in the samples from different management units. **C** The number of OTUs assigned to different families. **D** Average relative abundances of the families in the samples from different management units. **E** The number of OTUs assigned to different genera. **F** Average relative abundances of the genera in the samples from different management units. In **B**, **D**, and **F**, the length of color-coded bars represents the average relative abundance of the taxa in the samples in the indicated management units. Taxa with < 2% relative abundance in all the units were merged into the “Other/Unassigned” category

We identified a total of 4681 OTUs in the 90 fecal samples. These OTUs could be taxonomically assigned to 20 phyla, of which Firmicutes (2573/55.0%), Bacteroidetes (1291/27.6%), and Tenericutes (267/5.7%) included the highest number of the OTUs (Fig. 2A). While only Firmicutes (587,075/51.6% reads), Bacteroidetes (412,852/36.3% reads), Patescibacteria (53,402/4.7% reads), and Proteobacteria (16,379/1.4% reads) were observed in all 90 samples, they accounted for 94.1% of the total bacterial communities at the phylum level. Firmicutes had the highest average relative abundance in the samples from all the management units except hutch calves, where Bacteroidetes had the highest average relative abundance of 62.3% and Firmicutes only accounted for 33.4% (Fig. 2B).

At the family level, these OTUs could be assigned to 99 families, in which *Ruminococcaceae* (1441/34.9%), *Rikenellaceae* (500/11.6%), *Lachnospiraceae* (319/6.8%), and *Prevotellaceae* (273/8.5%) contained the highest number of the OTUs (Fig. 2C). Although only 12 families were detected in all 90 samples, they accounted for 992,097 (87.2%) reads of the total bacterial communities at this taxonomic level. *Ruminococcaceae* (397,075/34.9% reads) was the most dominant taxon in average relative abundance at this level, followed by *Rikenellaceae* (131,673/11.6% reads), *Prevotellaceae* (96,322/8.5% reads), *Lachnospiraceae* (77,143/6.8% reads), *Muribaculaceae* (70,454/6.2% reads) and *Saccharimonadaceae* (53,121/4.7% reads) (Fig. 2D).

At the genus level, 2169 (46.3%) of the 4681 OTUs could be assigned to 167 known genera, while the remaining 2512 OTUs (53.7%) could not be classified to known genera, thus annotated by 70 lowest known taxonomic ranks (family, order, etc.). These unclassified taxa might be novel bacteria in the cattle feces or not differentiable solely based on the hypervariable regions of 16S rRNA genes. Overall, the largest number of the OTUs were assigned to [f]*Ruminococcaceae* (588/11.9% OTUs), followed by *Rikenellaceae_RC9_gut_group* (288/6.15% OTUs), and *Ruminococcaceae_UCG-010* (250/5.3% OTUs) (Fig. 2E). While only 17 genera were consistently detected in the 90 samples, these commonly shared genera occupied 72.8% of the total bacterial communities at this taxonomic level. Most of the assigned genera had an average abundance of < 2% in the samples from the management units, while only 15 genera had a relative abundance ≥ 2% including [f] *Ruminococcaceae* (11.5%), *Ruminococcaceae_UCG-005* (8.8%), *Rikenellaceae_*RC9_gut_group (8.2%), [f]*Muribaculaceae* (6.2%), [f]*Lachnospiraceae* (5.2%), *Candidatus_Saccharimonas* (4.7%), [o] Bacteroidales (4.3%), *Bacteroides* (3.6%), *Ruminococcaceae_UCG-014* (3.5%), *Ruminococcaceae_UCG-013* (3.4%), *Christensenellaceae_R-7_group* (3.0%), *Ruminococcaceae_UCG-010* (2.6%), *Prevotellaceae_UCG-003* (2.5%), [f]*Prevotellaceae* (2.2%), and *Alistipes* (2.2%) (Fig. 2F).

### Alpha diversity of the cattle fecal bacterial communities in the management units

**Fig. 3:**
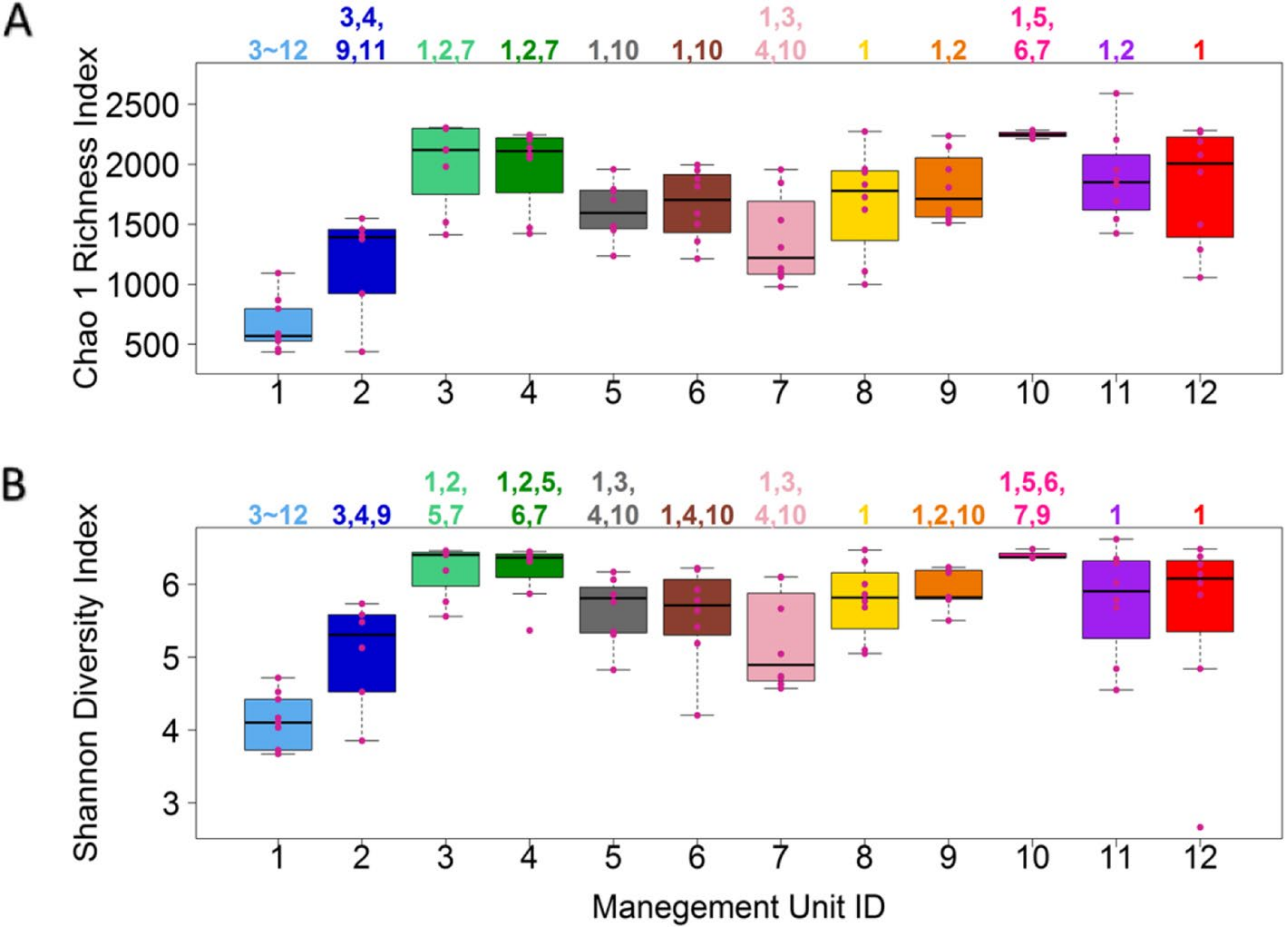
Diversity of fecal bacterial communities of cattle in the management units. Boxplots of Chao 1 richness index (**A**) and Shannon diversity index (**B**) of fecal bacterial communities of cattle in the management units (x-axis). Each violet-red dot represents the bacterial communities in a fecal sample, and management units are coded by distinct colors. The numbers above each box indicate the management units that are significantly different from the current one with the pairwise Wilcoxon test (*P* < 0.05)

We next compared the richness and evenness of the fecal bacterial communities of cattle in different management units in the production line using both Chao 1 richness index and Shannon diversity index. As shown in Fig. 3A and B, the fecal bacterial communities in pre-weaned calves (unit 1) fed primarily milk and housed in individual hutches (Table 1) had significantly lower Chao 1 and Shannon indexes than those in all other units, except for the post-weaned group-housed calves fed a solid diet (unit 2) (Kruskal Wallis, false discovery rate, FDR < 0.05), suggesting that the hutch calves generally had simpler fecal bacterial communities. Post weaned heifers (unit 2), while having no significant differences in bacterial communities compared with hutch calves (unit 1), consistently differed from breeding heifers (unit 3), springers (unit 4), and pregnant late lactation cows (unit 9), which might be related to the development of the rumen and these growing young cattle being full ruminants by the time they were bred.

### Similarity and difference of the fecal bacterial communities between management units

**Fig. 4:**
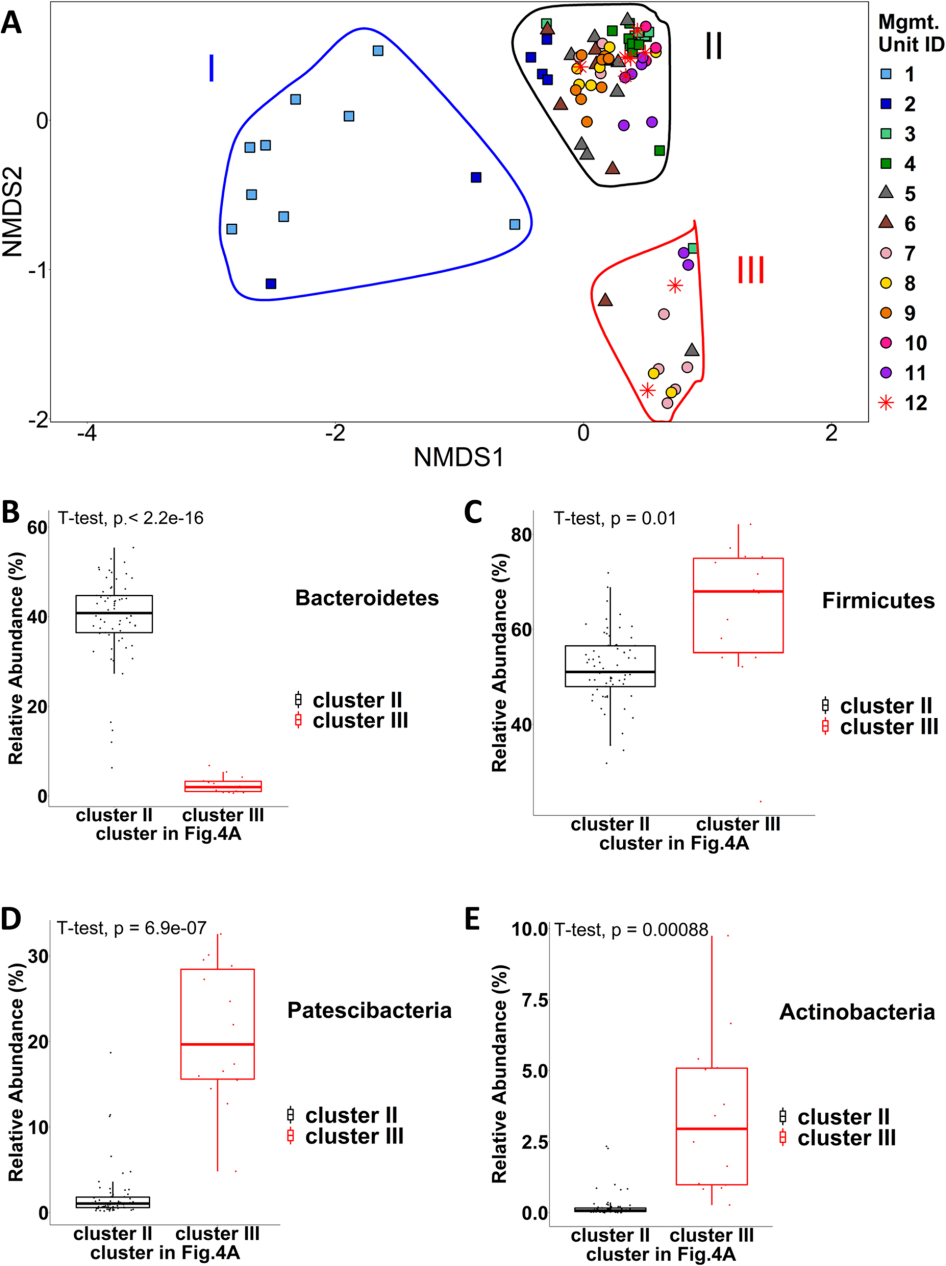
Similarity and difference of the fecal bacterial communities. **A** Non-metric multidimensional scaling (NMDS) plot of the Bray-Curtis distance for bacterial communities in different management units. Each point represents a fecal bacterial community and is colored by the management unit from which it was sampled. The communities are grouped into three clusters (I, II, and III) circled in blue, black, and red rings, respectively. Boxplot of four phyla’s relative abundances in clusters II and III for **B** Bacteroidetes **C** Firmicutes **D** Patescibacteria and **E** Actinobacteria

To further evaluate the similarities and differences of the bacterial communities between different management units, we calculated the *β*-diversity (Bray-Curtis distance) of the samples and visualized the results using non-metric multidimensional scaling (NMDS). As shown in Fig. 4A, the hutch calves’ samples (unit 1) were largely grouped to form a cluster (I), which separated from the samples from the other management units, with two post-weaned heifers’ (unit 2) samples included, suggesting that the fecal bacterial communities of hutch calves (unit 1) are similar to one another but largely different from those of cattle in the remaining management units. Interestingly, the samples from the other units form two rather compact yet distinct clusters II and III (Fig. 4A), suggesting the samples in each cluster have quite similar bacterial communities. Cluster II contains samples from all the management units except unit 1; in contrast, cluster III comprises samples only from units 3, 5, 6, 7, 8, 11, and 12, suggesting that non-neonatal cattle (from unit 2 to 12) may only have one of two types of rather uniform bacterial structures.

Moreover, we found that at the phylum level, the relative abundances of several phyla were significantly different between the samples in clusters II and III (Fig. 4B-E, t-test, *P* < 0.05). The samples in cluster II were high in Bacteroidetes (Fig. 4B), while samples in cluster III were high in Firmicutes (Fig. 4C), Patescibacteria (Fig. 4D), and Actinobacteria (Fig. 4E). There were also five additional phyla with low relative abundances showing significant differences between the two clusters (Additional file 3: Fig. S2).

### Shared and unique taxa at the genus level in the management units

For the 237 taxa at the genus level (167 assigned to known genera and 70 assigned to higher taxonomic ranks), 112 of them had the average relative abundance of 0.1% or greater in at least one management unit. Among these 112 genera, 70 were observed in all management units while the remaining 42 genera existed in a varying number of management units. Particularly, there were several genera largely detected only in management unit 1but barely seen in other units, suggesting these taxa may play important roles in the early stage of life for the cattle. For example, 10 genera, as shown in Table 2 listed by the relative abundance from high to low, were observed majorly in unit 1 but sparsely seen in other units. In contrast, we found *Prevotellaceae_UCG-004*, *dgA-11_gut_group*, *Caproiciproducens*, and an unassigned genus ([f]*p-2534-18B5_gut_group*) missing in animals from unit 1, but in all other 11 management units (Table 2).

**Table 2:** Number of occurrences and taxonomic assignment at the genus level for taxa that were either majorly detected in unit 1 or missing in unit 1 but observed in other units

Avg. No. of Reads in Unit 1	Avg. No. of Reads in Unit 2	Sum of Avg. No. of Reads in other 10 Units	Phylum	Family	Genus
Genera majorly observed in unit 1					
330 (2.6%)	4	2	Bacteroidetes	*Prevotellaceae*	*Prevotella_*9
157 (1.2%)	0	0	Bacteroidetes	*Prevotellaceae*	*Prevotella_*2
102 (0.8%)	0	3	Bacteroidetes	*Prevotellaceae*	*Prevotella*
97 (0.8%)	10	5	Clostridiales	*Ruminococcaceae*	*Faecalibacterium*
87 (0.7%)	0	1	Proteobacteria	*Burkholderiaceae*	*Sutterella*
86 (0.7%)	9	0	Bacteroidetes	*Tannerellaceae*	*Parabacteroides*
74 (0.6%)	2	0	Fusobacteria	*Fusobacteriaceae*	*Fusobacterium*
72 (0.6%)	17	0	Firmicutes	*Ruminococcaceae*	*Subdoligranulum*
54 (0.4%)	4	0	Firmicutes	*Ruminococcaceae*	*Oscillospira*
86 (0.7%)	9	0	Bacteroidetes	*Tannerellaceae*	*Parabacteroides*
15 (0.1%)	0	6	Erysipelotrichales	*Erysipelotrichaceae*	*Erysipelotrichaceae_*UCG-004
*Genera missing in unit 1 but observed in other units*					
0	31	1581	Bacteroidales	*Prevotellaceae*	*Prevotellaceae_UCG-004*
0	1	1196	Bacteroidales	*p-2534-18B5_gut_group*	Unassigned, annotated as [f]*p-2534-18B5_gut_group*
0	16	905	Bacteroidales	*Rikenellaceae*	*dgA-11_gut_group*
0	2	401	Clostridiales	*Ruminococcaceae*	*Caproiciproducens*

### Dynamics of bacterial communities between the management units

**Fig. 5:**
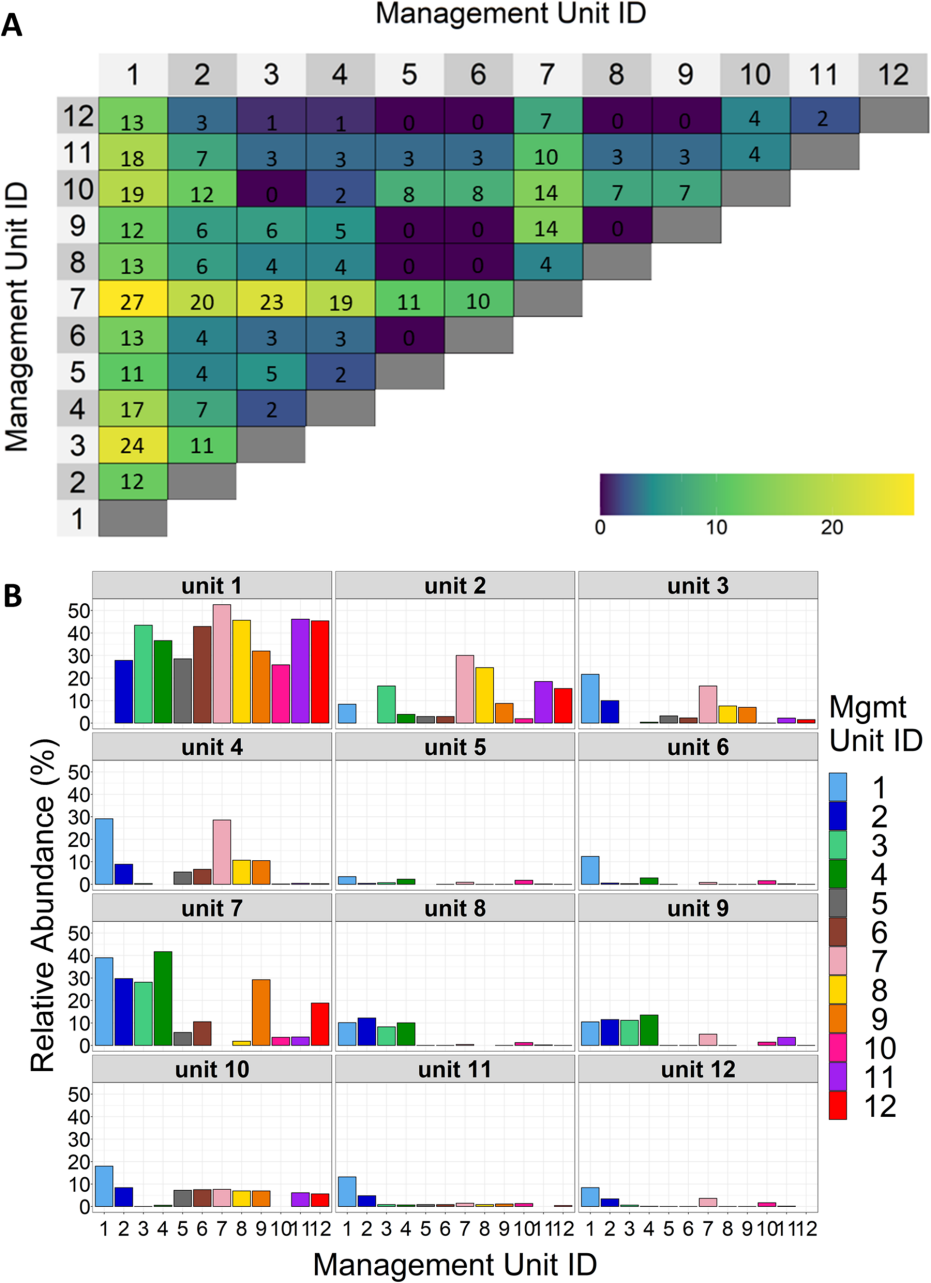
Significantly different taxa at genus level between all pairs of management units. **A** Number of genera significantly different between each pair of the management units. **B** Relative abundance of significantly different genera between each pair of the management units

From the core bacteria, the 112 genera with an average minimal abundance of 0.1% in at least one management unit), we found 56 of them displayed significantly different abundances in the samples from the 12 management units (ANOVA, FDR < 0.05). We further summarized how each of these 56 genera was significantly different in their composition between different pairs of management units (Tukey HSD, *P* < 0.05, Fig. 5A and B). For example, unit 1 had a varying number of significantly different genera with all other 11 units, from 11 with unit 5 to 27 with unit 7 (Fig. 5A). As shown in Fig. 5B, the relative abundances of these significantly different genera between unit 1 and other units were in the range of 25.9 and 52.6%. Interestingly, there is no significantly different genus between any pair of management units 5, 6, 8, and 9, suggesting the types and relative abundances of the genera were similar in these four units.

### Synergistic and antagonistic relationships

**Fig. 6:**
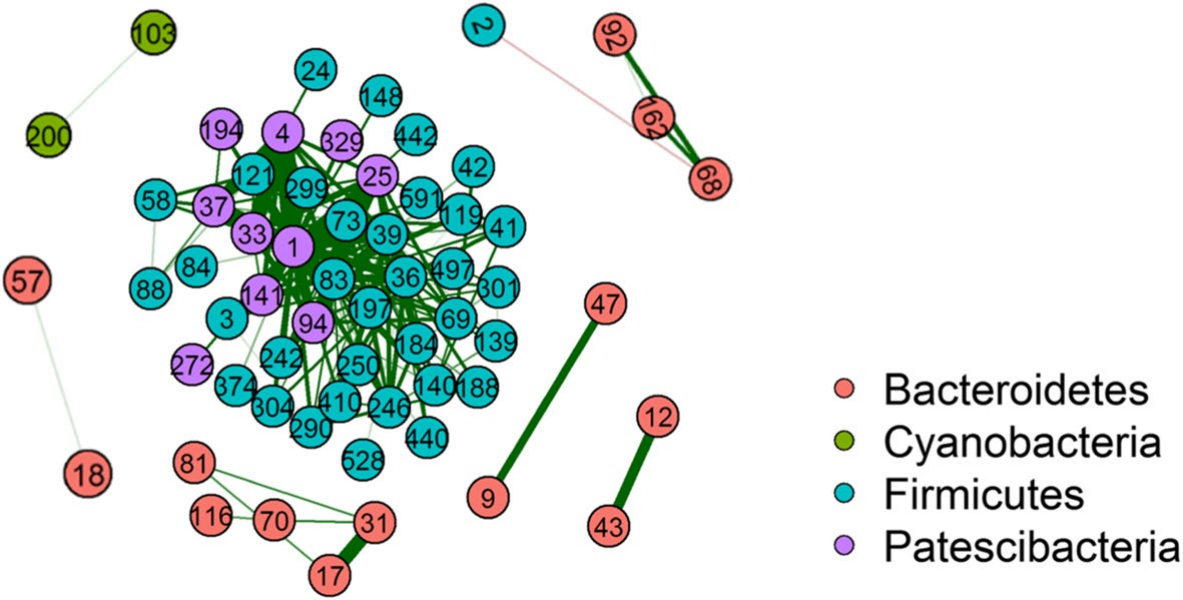
Strong correlations in the core bacteria. Each node is an OTU and the number within represents the OTU ID. Nodes are colored based on their taxonomic assignment at the phylum level. The edges in green and red indicate positive and negative correlations

Bacteria thriving in a community may interact with one another synergistically or antagonistically. To reveal such potential interactions between different bacteria, we calculated the correlations of the core bacteria using the SparCC (Sparse Correlations for Compositional data) algorithm [16]. By setting ±0.7 as the threshold values for strong positive and negative correlations and pseudo *p*-values < 0.05 as the significant correlations, we found 61 OTUs that had strong correlations with at least one other OTU (Fig. 6; Additional file 4: Supplementary file 2; Additional file 5: Supplementary file 3). Most of these strong correlations are positive, while only one pair between *Ruminococcaceae_UCG-005* and *Bacteroides* is negative (SparCC = − 0.70). In Fig. 6, all the strong correlations are shown within the networks. From the 181 strong correlations, 117 are between the OTUs assigned to the same phylum, indicating that strong correlations are more likely to be within the same phylum than those from different phyla.

### Functional prediction of the bacterial community

To further explore the fecal bacteria of the dairy cattle, we predicted the functions of the bacterial community using a marker gene based tool Tax4Fun2 [17]. Compared to the initial version Tax4Fun, Tax4Fun2 has higher accuracy, allows users to customize the reference genomes, and integrates a multifunctional redundancy evaluation [17, 18].

**Fig. 7:**
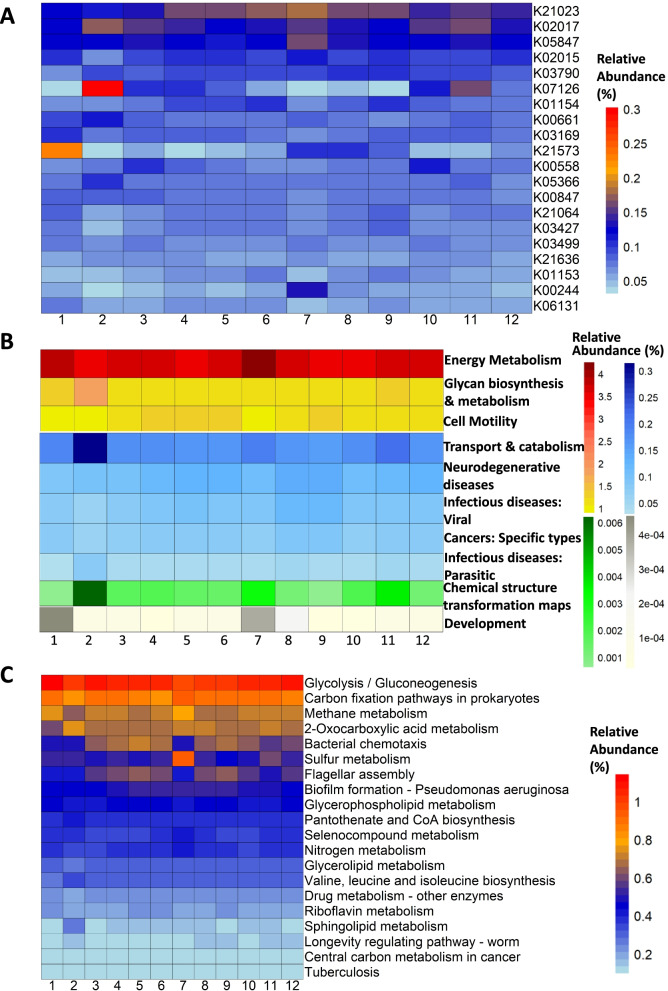
Significantly differential orthologs and pathways in the KEGG database predicted using Tax4Fun2. **A** Top 20 the most abundant significantly differential KOs. **B** Significantly differential level 2 pathways. **C** Top 20 the most abundant significantly differential level 1 pathways. Rows represent KOs/pathways and columns represent management units. Subplots were arranged by the relative abundance from high to low

A total of 7726 Kyoto Encyclopedia of Genes and Genomes (KEGG) Orthologs (KOs) were predicted in the identified bacterial microbiota in the samples while no single KO had a relative abundance higher than 2% in any sample (Additional file 6: Supplementary file 4). Among them, 4001 (51.8%) KOs were present in all 90 samples, indicating these molecular functions were essential for the cattle regardless of age, housing, and production status such as K00001 (alcohol dehydrogenase) that mediates the oxidation and reduction of ethanol [19]. Interestingly, some KOs were only present in the samples from one management unit. For instance, a collection of 9 KOs (K04340, K14194, K16046, K16227, K18254, K18611, K18906, K20218, and K21329) were only observed in some samples of unit 1, though at very low abundances (Additional file 6: Supplementary file 4). These KOs might be involved in the food digestion of hutch calves. For instance, K18611 (4-pyridoxic dehydrogenase) is an enzyme that degrades vitamin B6, which is contained in milk, the primary diet for hutch calves housed in unit 1 [20]. There were 982 (12.7%) significantly differential KOs (ANOVA, FDR < 0.05) and the top 20 most abundant ones were shown in Fig. 7A.

From the pathway perspective, Tax4Fun2 predicted 6, 45, and 239 pathways at KEGG level 3, 2, and 1, respectively. Metabolism (70.4–72.0%) was the most abundant pathway at level 3 in every management unit, followed by Environmental Information Processing (12.0–13.2%), Cellular Processes (6.1–6.7%), Genetic Information Processing (5.3–5.7%), Human Diseases (2.8–3.1%), and Organismal Systems (1.2–1.3%, Additional file 7: Supplementary file 5). Further analysis revealed 10 significantly differential pathways at level 2, with the energy metabolism pathway being the most abundant (Fig. 7B). However, the abundances of these pathways combined were only 6.6% on average. At level 1, there were 68 significantly differential pathways, and the most abundant 20 of them were shown in Fig. 7C. Again, energy metabolism related pathways are most abundant, such as glycolysis/gluconeogenesis, carbon fixation, and methane metabolism.

## Discussion

In modern dairy management, it is not uncommon to house animals in different management units based on their ages, nutrition requirements, reproductive status, lactation status, production, and management styles [21]. Such a management system optimizes milk production and management but brings challenges profiling and analyzing the bacterial communities in the GIT because the bacterial communities can be influenced by numerous factors including, but not limited to, diet, animal physiology and status, the farm environment, geographic location, antimicrobial use, and management practices of the production lifecycle [6, 12, 22–25]. For instance, the bacterial communities of cattle housed individually were less likely to be affected by other cattle compared with those housed together in open pens. Another example is that cattle with diseases may be treated with antimicrobial drugs that may directly change the balance of the bacterial communities.

In this study, we sought to characterize the composition, diversity, and dynamics of the fecal bacteria in different management units over the production lifecycle of dairy cattle on a California dairy farm. We used paired-end sequencing of the V3/V4 hypervariable regions of the 16S rRNA genes to profile the bacterial communities in 90 fecal samples collected from 90 cattle in the 12 management units. Our results suggest high similarity between any two of the management units 5, 6, 8, and 9, but show significant differences in most of the other management unit pairs in terms of richness, evenness, and structure of the gut bacterial communities at the genus level.

Overall, consistent with several earlier studies [6, 7, 12, 13, 22], our results reveal Firmicutes and Bacteroidetes were the two most dominant taxa at the phylum level, distantly followed by several other phyla. Specifically, our results show that, on average, the Firmicutes and Bacteroidetes represented 51.6 and 36.3% of the total communities, respectively. Interestingly, the relative abundance of Bacteroidetes was lower in cows immediately before and after calving (within two weeks from calving) in units with fresh multiparous cows (18.7%) and close up dry cows (23.2%), while the relative abundances were in the range of 29.4 and 62.2% in other units (Fig. 2B).

For some genera, as Mao et al. [7] demonstrated, *Prevotella* had a lower abundance in samples taken from the small and large intestines, but higher in the forestomach, such as the rumen. In our work, the relative abundance of *Prevotella* was only 0.07%. However, in one of the hutch calves’ (unit 1) samples, we observed *Prevotella* had a relative abundance of 7.16% (905 reads) while the number of *Prevotella* reads in the other 89 samples was in the range of 0 to 11. We also found in this sample, *Ruminococcus_1* had a relatively high abundance (5.56%, 703 reads) while this genus was in the range of 0 to 115 reads in other samples. So potentially there is some synergistic relationship between *Prevotella* and *Ruminococcus_1* and the interaction prevent *Prevotella* from being degraded or eliminated when going through the GIT. Such interaction could also be related to the diet of hutch calves being primarily milk compared to all the other units fed a solid diet. Moreover, feeding waste milk from the hospital pen or fresh cow pen to calves is a common practice in the dairy industry and was done on this dairy at the time of sampling. Waste milk commonly contains antibiotic residues of varying concentrations as it is collected from milking cows in the hospital being treated with antibiotics or awaiting clearance during their withdrawal period before rejoining the pens where milk is harvested for human consumption.

It is noticeable that bacterial communities in hutch calves were significantly different from those in other management units except post-weaned heifers (Figs. 2, 3, 4A, 5). These differences confirmed the earlier findings that the enteric microbiota in neo-natal calves were different from adult cows and the bacterial communities underwent a dramatic change during the development at an early age [9, 10, 23]. Intriguingly, bacteria from the samples in post-weaned heifers displayed similar patterns with the communities from the adult cattle, predominately Firmicutes; but at the same time, they showed no significant difference from those of hutch calves. As the post weaned heifers were aged from approximately 2 months (70 days on average) to 13-month old, the microbiota changes were still ongoing towards the bacterial profiles in adults during this transition period. Using the same set of samples, we have previously found that *E. coli* from hutch calves exhibited a wider spectrum of resistance to antimicrobial drugs compared to bacteria from other units [21]. Currently, it largely remains undetermined concerning the roles of bacterial communities on antimicrobial resistance of specific bacterial species, however, in future studies, it will be interesting to assess such roles, for example, resistomes on phenotypes of antimicrobial resistance.

For the samples from non-neonatal cattle (from unit 2 to unit 12), we did not find any evidence supporting that the bacterial communities could be clustered based on the management unit membership. This demonstrated that the management unit itself is not a determinate force for the structure of the bacterial communities. Instead, as shown in Fig. 4A, we did observe distinct patterns where most of our samples from non-neonatal cattle formed two clusters (II and III), implying they had two major different structures. As we looked deeper, we found that at the phylum level, the relative abundances of several phyla became very different between the two clusters (Fig. 4B-E). For example. in cluster III, the mean and median of Patescibacteria relative abundance were 20.9 and 19.7% while those values in cluster II were only 1.9 and 1.1%, respectively. What caused the differences in Patescibacteria in various samples still needs future research, but the potential interactions between some taxa classified as Firmicutes and Patescibacteria seem to prevent Patescibacteria from decreasing. It has been reported that the rumen bacteria in the 1st and 2nd lactations, which overlapped with some of our management units, are dynamic yet similar, and the samples from the two lactations cannot be separated by the lactation cycle in PCA visualization [8]. This demonstrated that the bacterial structure might undergo further shaping going through the GIT (their rumen samples versus our fecal samples) or the formed patterns were just a case-sensitive phenomenon.

Pitta et al. reported that significant bacterial population change was observed during the transition from 21 days before calving to 21 days after calving in uniparous and multiparous cows, respectively [26]. This shift period corresponded approximately to our management units 4 and 5 for uniparous cows and units 10 and 11 for multiparous cows. However, we did not see this significant shift as demonstrated earlier [26]. The only significant difference we observed was Shannon diversity between units 4 and 5. This could be due to the different sites of the GIT from which the samples were taken (their rumen samples versus our fecal samples). It is also possible that this was due to the four management units in our study not being strictly narrowed to 21 days prior and post-calving. As the dairy farm environment is dynamic, it is not surprising that most taxa were shared by cattle housed in different management units.

Measuring potential bacterial interactions was challenging and barely reported in dairy cattle studies. The classic correlation methods had their limitations when applied to genomic data such as 16S rRNA gene sequencing data which are sparse and compositional. SparCC as a method that negates the negative correlation bias of compositional data [27] and identifies true association missed by others [28], was used here as a way of evaluating bacterial interactions. In general, we identified 180 strong positive correlations and 1 strong negative correlation between 61 OTUs (Fig. 6). These co-occurrent OTUs may tend to share the same habitats and perform similar functions, as most of these co-occurrent patterns spotted between OTUs were in the same phyla (Fig. 6).

## Conclusions

In this study, we profiled the structure and dynamics of gut bacterial communities from cattle in 12 independent yet inter-connected management units on a modern California dairy farm. To the best of our knowledge, this is the first study that describes the bacterial communities across all management units and reveals the structures of gut microbial communities to each of the well-defined management units. We analyzed the changes in gut microbial communities across production systems. Though the fecal microbiota were similar in 4 of the management units, they showed significant changes between others. It is confirmed that microbial ecology underwent dramatic changes in the early days of life, as evidenced by the significantly different bacteria in hutch calves from other adult cattle and revealed dynamics of the bacterial abundance in the later stages of the production lifecycle. Moreover, we identified at least 10 genera that were detected only in hutch calves but were absent in all the other cattle in other units. These genera might play crucial roles in the early establishment and development of the GIT. We also dissected potential interactions among gut bacterial groups, mostly from the species in the same phylum.

## Methods

### Sample collection and study herd

On a single day in June 2016, a total of 90 fecal samples were collected from 90 individual cattle in 12 management units (Fig. 1, Table 1) from a dairy herd in the Central Valley of California, USA. Cattle in each of these management units were identified based on convenience sampling. Trained study personnel collected the fecal samples manually from the rectum of cattle using standard veterinary protocols. Other information such as the sampling population in each management unit can also be found at Li et al. [21]. These samples were also used for an antimicrobial resistance study that has been published by Li et al. [21]. An aliquot of each sample shipped in refrigerated conditions to Dr. Su’s laboratory at the Department of Bioinformatics and Genomics, the University of North Carolina at Charlotte for 16S rRNA gene sequencing.

### Illumina MiSeq sequencing

DNA from stool samples was extracted with the Qiagen DNA Stool kit following the manufacturer’s instructions. Two steps of Polymerase Chain Reaction (PCR) procedures were used to generate amplicons from the 16S RNA genes for sequencing. The first-round PCR was to target V3/V4 regions of 16S rRNA genes with the forward primer: 5′- CCTACGGGNGGCWGCAG and the reverse primer: 5′- GACTACHVGGGTATCTAATCC. This step was done with the KAPA Biosciences HiFi PCR kit and additional BSA. The protocol consists of initial denaturation at 95 °C for 3 min, followed by 25 cycles of denaturation (90 °C for 30 s, 55 °C for 30 s, and 72 °C for 30 s), and final elongation at 72 °C for 5 min. The PCR products were cleaned up with Ampure XP beads. The second-round PCR was performed with Nextera XT index Primers and sequencing Adaptors with the following setting: initial denaturation at 95 °C for 3 min, followed by 8 cycles of denaturation (90 °C for 30 s, 55 °C for 30 s, and 72 °C for 30 s), and final elongation at 72 °C for 5 min. The PCR products were cleaned up with Ampure XP beads and paired-end sequenced (2 × 300 bp) on an Illumina MiSeq platform at the University of North Carolina at Charlotte.

### OTU table construction

Primers with raw sequences were removed by Cutadapt [29]. We performed quality control using DADA2’s “filterAndTrim” function with “trancLen” equal to 200 bp for forward reads and 150 bp for reverse reads based on quality profiles. Technical error rate learning was performed with all the sequences in the samples. Sample inference was performed by the “dada” function with the setting optional parameter “pool = TRUE.” Paired-end reads merger in DADA2 resulted in approximately 50% loss of sequences, thus only forward reads were used in this study. OTU table and chimera removal were implemented with default parameters. We used the “assignTaxonomy” function that provided a native implementation of the RDP Classifier [30] with minimum bootstrap confidence of 80 to assign taxonomy from the phylum level to the genus level to each OTU. SILVA database release 132 [31] was used as the reference database.

### Function prediction

Molecular function prediction was done using Tax4Fun2 with the default reference library. The functional profiles were generated with the built-in functions “runRefBlast” and “makeFunctionalPrediction” with default settings.

### Statistical analysis

Rarefying was performed by the “single_rarefaction” function with the minimum number of reads of all samples, which is equal to 12,637 in QIIME [32]. We used alpha diversity, including Chao 1 index which evaluates richness, and Shannon index which evaluates diversity to measure the within-sample diversity. Differences in Chao 1 index and Shannon index in different units were assessed by the Kruskal Wallis test with the Benjamini-Hochberg (BH) correction for multi-comparisons, and pairwise management units’ comparisons were performed by pairwise Wilcoxon test. The Bray-Curtis distance matrix was employed to perform beta diversity analysis. Visualization was done by a non-metric multidimensional scaling plot. SparCC correlations were calculated by FastSpar [33], a C++ implementation of SparCC algorithm (100 bootstrap samples were generated for pseudo *p*-value calculation). R software [34] and R packages ggplot2 [35], vegan [36], superheat [37], ggpubr [38], and qgraph [39], were used for calculation and visualization.

## Supplementary Information

**Additional file 1: Figure S1.** Rarefaction curves showing “Number of Observed OTUs” as a function of “Number of Sequencing Reads” for each sample.

**Additional file 2: Supplementary file 1.** Normalized OTU table. The table includes the information of OTU ID, abundance, and taxonomic assignment for each OTU.

**Additional file 3: Figure S2.** Boxplot of five additional phyla with low relative abundances showing significant difference between clusters II and III in Fig. 4A.

**Additional file 4: Supplementary file 2.** SparCC table. Table of calculated SparCC correlations and pseudo *p*-values for 61 OTUs that had strong correlations with at least one other OTU.

**Additional file 5: Supplementary file 3.** Taxonomic table. Table of taxonomic classification of 61 strongly correlated OTUs.

**Additional file 6: Supplementary file 4.** The table of function predictions generated by the Tax4Fun2 program.

**Additional file 7: Supplementary file 5.** The table of pathway predictions generated by the Tax4Fun2 program.

## Data Availability

All DNA sequences have been deposited in NCBI’s Sequence Read Archive (SRA) with the accession number PRJNA607283. Direct link: https://www.ncbi.nlm.nih.gov/sra/?term=PRJNA607283

## Abbreviations

GIT: Gastrointestinal tract
NGS: Next-generation sequencing
OTU: Operational taxonomy unit
NMDS: Non-metric multidimensional scaling
SparCC: Sparse correlations for compositional data
KEGG: Kyoto encyclopedia of genes and genomes
KO: KEGG ortholog
