# Supplementary material for: Dynamic changes in fecal bacterial microbiota of dairy cattle across the production line
Source: BMC Microbiol. 2022 May 14;22:132. doi: 10.1186/s12866-022-02549-3 (PMC9107139; doi:10.1186/s12866-022-02549-3)
Supplement: Supplementary file 1 — Additional file 1: Figure S1. Rarefaction curves showing “Number of Observed OTUs” as a function of “Number of Sequencing Reads” for each sample. [file 12866_2022_2549_MOESM1_ESM.docx]

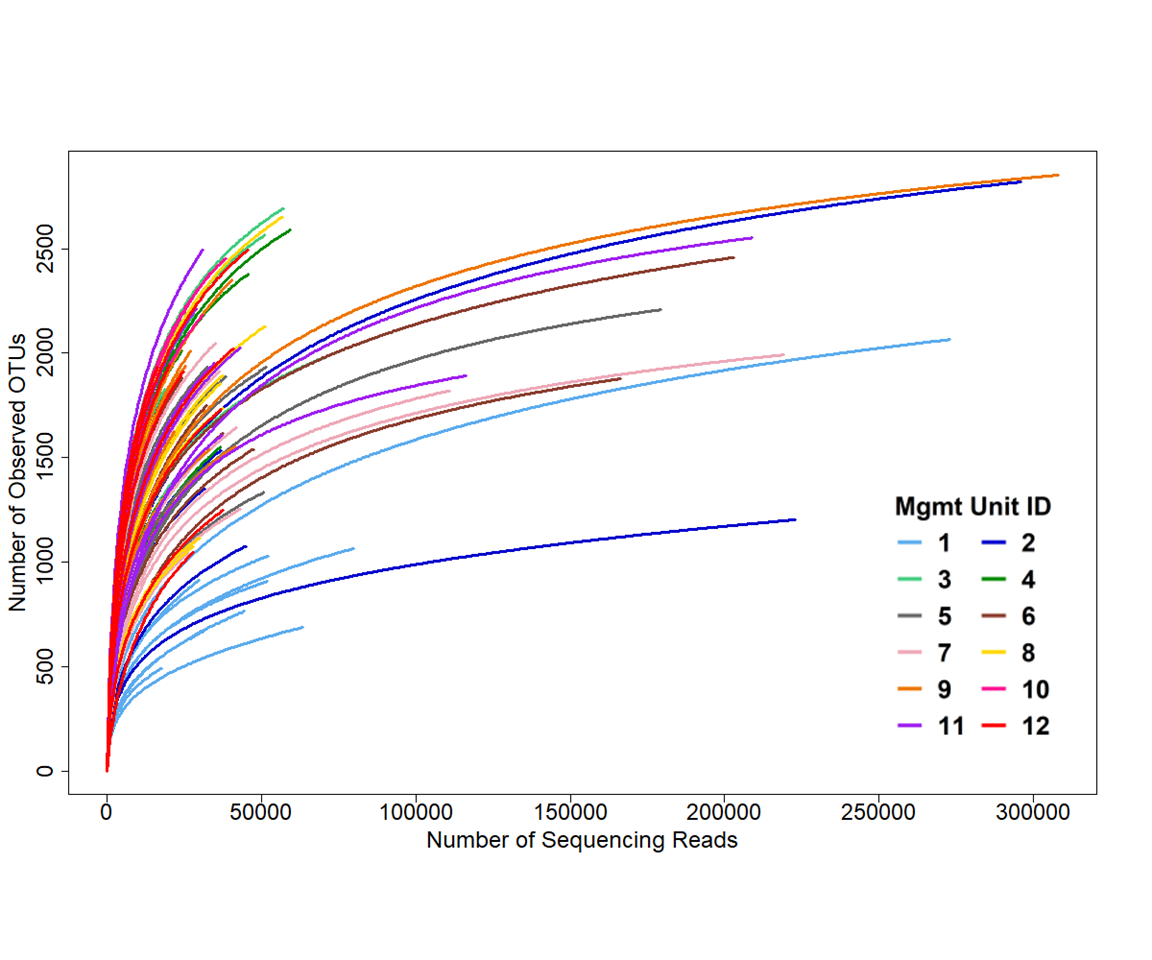


**Figure S1.** Rarefaction curves showing “Number of Observed OTUs” as a function of “Number of Sequencing Reads” for each sample. The curves were colored by management unit.
