# Supplementary material for: Dynamic changes in fecal bacterial microbiota of dairy cattle across the production line
Source: BMC Microbiol. 2022 May 14;22:132. doi: 10.1186/s12866-022-02549-3 (PMC9107139; doi:10.1186/s12866-022-02549-3)
Supplement: Supplementary file 3 — Additional file 3: Figure S2. Boxplot of five additional phyla with low relative abundances showing significant difference between clusters II and III in Fig. 4A. [file 12866_2022_2549_MOESM3_ESM.docx]

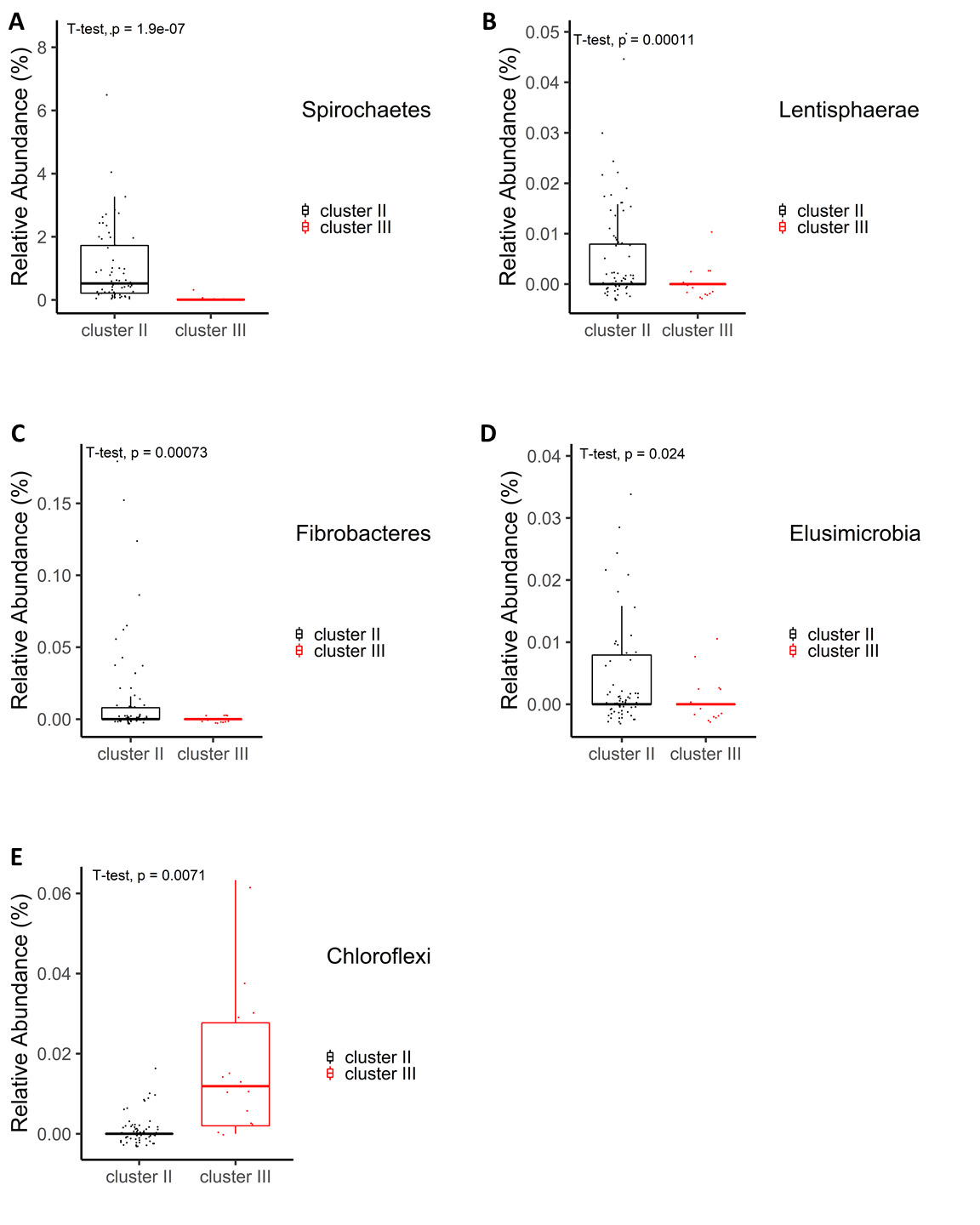


**Figure S2.** Boxplot of five additional phyla with low relative abundances showing significant difference between two clusters in Fig.4A. (A) Spirochaetes (B) Lentisphaerae (C) Fibrobacteres (D) Elusimicrobia (E) Chloroflexi.
